# Porcine Leydig cells: central regulators of boar reproductive development and fertility

**DOI:** 10.1093/jas/skag215

**Published:** 2026-07-10

**Authors:** Amy T Desaulniers, Caitlin E Ross, Brett R White

**Affiliations:** School of Veterinary Medicine and Biomedical Sciences, University of Nebraska–Lincoln, Lincoln, NE 68583, United States; School of Veterinary Medicine and Biomedical Sciences, University of Nebraska–Lincoln, Lincoln, NE 68583, United States; Department of Animal Science, University of Nebraska–Lincoln, Lincoln, NE 68583, United States

**Keywords:** boar, fertility, Leydig cell, steroidogenesis, testosterone, testis

## Abstract

Leydig cells within the interstitium of the testis produce the sex steroids (e.g., testosterone and 17β-estradiol), which are essential to male fertility. Unlike most species, pigs have three distinct phases of Leydig cell development and function. The first transient phase occurs during early fetal life, leading to the production of testosterone, which is critical for sex differentiation and early testicular development. The second transient phase in Leydig cell development occurs during the perinatal/neonatal period; during this developmental window, gonadal steroids further masculinize the brain and regulate testicular development. The final phase of Leydig cell development begins prior to puberty, between 100 and 130 d of age. The adult Leydig cell population remains active throughout adulthood. Steroidogenesis within adult Leydig cells is classically stimulated by luteinizing hormone synthesized and released from the anterior pituitary gland. Porcine Leydig cells produce androgens, as well as relatively high concentrations of estrogens, via the Δ5 pathway. Steroidogenesis is reliant on both acute (mobilization of cholesterol and activity of steroid acute regulatory protein [StAR]) and chronic (upregulation of gene expression, organelle expansion, and cell proliferation) responses. Many proteins and enzymes are involved in driving steroidogenesis within Leydig cells, including StAR, CYP11A1, CYP17A1, 3β-HSD, 17β-HSD, and CYP19A3. The production of gonadal steroids by adult Leydig cells is essential for numerous reproductive processes, including spermatogenesis, sperm maturation within the epididymis, accessory sex gland function, and libido. Thus, Leydig cells are critical regulators of boar fertility.

## Teaser

Boar fertility is a critical determinant of reproductive efficiency in swine production and is fundamentally dependent on testosterone synthesis by testicular Leydig cells. This review summarizes the developmental phases of porcine Leydig cells and explains how their steroidogenic function supports the key reproductive processes that underlie fertility in boars.

## Introduction

Boar fertility is a crucial component of swine production. Approximately 27,000 boars in the United States (Knox 2012) affect conception rates, farrowing rates, and litter sizes (Swierstra and Dyck 1976) for 11.8 million litters annually (USDA-NASS 2025) due to the nearly exclusive use of artificial insemination (AI) (Flowers 2015). An average U.S. boar sires 8,398 progeny per year (Safranski 2008), which is approximately 327-fold more offspring than a sow (26 piglets/year) (USDA-NASS 2025). Consequently, the boar disproportionately impacts reproductive success, genetic progress, and profitability of the U.S. swine industry (Flowers 2015). In 1994, only 15% of the swine industry utilized AI, but now greater than 90% of pig farms rely exclusively on AI (Flowers 2015). The success of AI is highly dependent on selecting boars with high libido and semen quality (Okere et al. 2005), processes that both rely on testosterone production from Leydig cells within the testis (Zirkin and Papadopoulos 2018).

Testosterone is a critical regulator of male fertility (Zirkin and Papadopoulos 2018). In mammals, the production of testosterone is classically regulated via the hypothalamic–pituitary–gonadal (HPG) axis (Zirkin and Papadopoulos 2018). Specifically, gonadotropin-releasing hormone (GnRH-I) from the hypothalamus binds to its receptor (GnRHR-I) on gonadotropes within the anterior pituitary gland, eliciting the synthesis and release of luteinizing hormone (LH), which subsequently stimulates Leydig cells within the testis to produce and secrete testosterone (Zirkin and Papadopoulos 2018). During the lifespan of a boar, testosterone is produced by three sequential populations of Leydig cells for distinct purposes including sexual differentiation, masculinization of the brain, sperm production, accessory sex gland function, and sexual behavior (Lunstra et al. 1986). Thus, the importance of Leydig cell function to boar fertility is unequivocal. This review examines the development and function of porcine Leydig cells, with a particular focus on the role of testosterone across the lifespan.

## Porcine Leydig cell development

### Fetal Leydig cells

In the pig, fetal Leydig cells begin to differentiate at approximately 28 days post-coitus (DPC) and express the steroidogenic enzyme, cytochrome P450 family 17 subfamily A member 1 (CYP17A1) (McCoard et al. 2001, 2002). Leydig cells are visible within the interstitium at 30 DPC, coinciding with the first detectable concentrations of testosterone in fetal serum and amniotic fluid (Ford et al. 1980). At this time (30 DPC), fetal Leydig cells also begin making insulin-like 3 (INSL3), which stimulates transabdominal testicular descent (Nef and Parada 1999; Vernunft et al. 2016). By 35 DPC, Leydig cells have increased in size, consistent with peak steroidogenic activity (Ford et al. 1980). Moreover, Leydig cells become densely packed with smooth endoplasmic reticulum (sER) and have elevated 3β-hydroxysteroid dehydrogenase (3β-HSD) activity (Ford et al. 1980). Relative testis weight (testis weight/body weight) is also elevated during fetal Leydig cell differentiation (Van Straaten and Wensing 1978).

**Figure 1:**
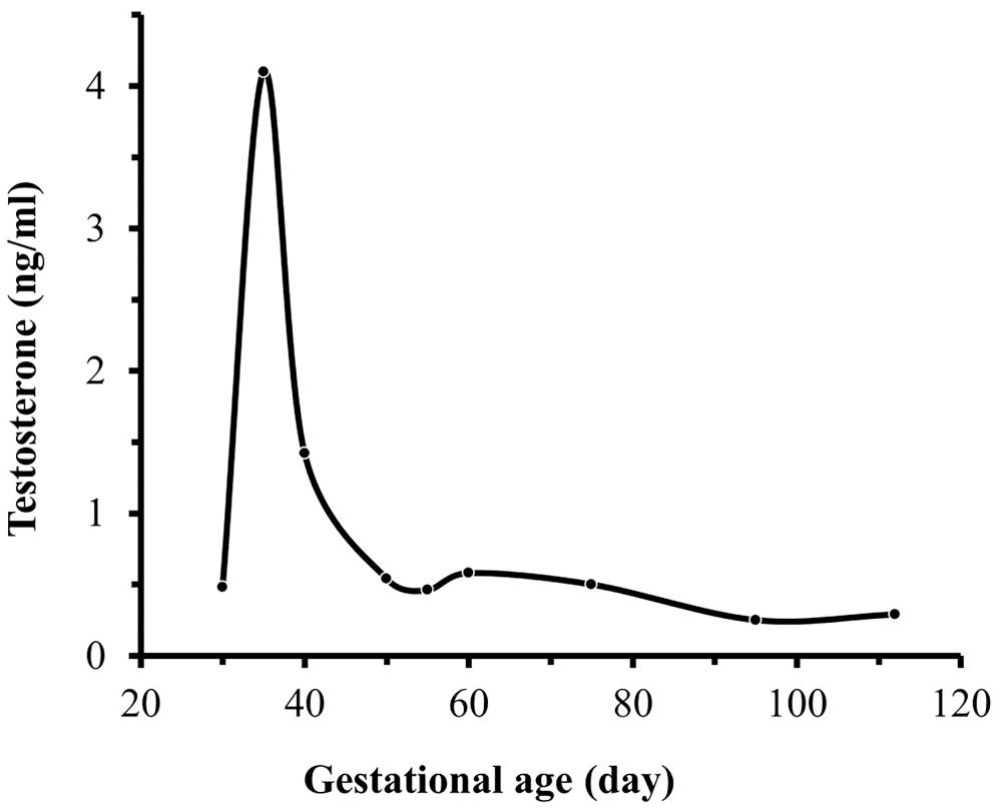
Testosterone production in the fetal boar. Serum testosterone concentrations were measured in fetal boars across gestation. Data replotted from Ford et al. (1980).

Testosterone concentrations in fetal serum peak (∼4 ng/mL) at 35 DPC and decline to ∼1.5 ng/mL by 40 DPC (Figure 1) (Ford et al. 1980). Concentrations further decline to ∼0.5 ng/mL by 50 DPC, likely due to decreased fetal Leydig cell numbers as well as cellular involution (Ford et al. 1980). A similar pattern was observed for testosterone levels in amniotic fluid (Ford et al. 1980). From 60 to 85 DPC, only a few immature Leydig cells are present within the testis, probably the remnants of fetal Leydig cells (Colenbrander et al. 1977). These cells are characterized by a small cell volume and nuclear diameter (5 µm), as well as undetectable 3β-HSD production (Van Straaten and Wensing 1978). Indeed, serum testosterone concentrations are low at this stage (Colenbrander et al. 1977) and cell proliferation is stagnant; the percentage of Leydig cells within the testis does not change between 49 and 95 DPC (Van Straaten and Wensing 1978).

Interestingly, fetal Leydig cells are not dependent on LH from the anterior pituitary gland (van Vorstenbosch et al. 1984), as the hypothalamus has not yet differentiated (Danchin and Dubois 1982). Immunoreactive GnRH neurons begin developing in the pig brain around 40 DPC, but do not reach the median eminence until 60 DPC (Danchin and Dubois 1982). Capillaries of the median eminence are not apparent until 70 DPC (Danchin and Dubois 1982) and LH is only first evident in serum of the male fetus at about 80 DPC (Colenbrander et al. 1977). In fact, fetal Leydig cell development occurs normally in decapitated embryos (van Vorstenbosch et al. 1982).

### Perinatal Leydig cells

The second phase in Leydig cell development begins at approximately 95 DPC (Colenbrander et al. 1977), whereby immature Leydig cells begin to develop; total weight, volume percentage, cell volume and nuclear diameter of these Leydig cells increase (Van Straaten and Wensing 1978). Moreover, 3β-HSD and 17β-hydroxysteroid dehydrogenase (17β-HSD) activity begin to increase (Van Straaten and Wensing 1978). In addition to the development of these immature (pre-existent) Leydig cells, mesenchymal cells begin differentiating into perinatal Leydig cells as well (Van Straaten and Wensing 1978; Klonisch et al. 2004). Interestingly, the latter population of Leydig cells develop in the peritubular regions, surrounding the immature seminiferous tubules (sex cords), whereas the former population is located within the interstitium (Van Straaten and Wensing 1978).

Between postnatal days (PND) 14–21, maximal development of perinatal/neonatal Leydig cells is achieved (Van Straaten and Wensing 1978). Both intertubular and peritubular Leydig cells are distinguishable; however, intertubular Leydig cells are most prominent (Van Straaten and Wensing 1978). At this stage, intertubular Leydig cells exhibit maximal cell and nuclear volumes, a characteristically differentiated appearance, abundant 17β-HSD activity, and low 3β-HSD activity. Peritubular Leydig cells are also visible but they are significantly smaller than intertubular Leydig cells, probably because they began to differentiate later (Van Straaten and Wensing 1978). Indeed, at PND 21, peritubular Leydig cells display many of the same characteristics as newly differentiated intertubular Leydig cells (e.g., high 3β-HSD and low 17β-HSD activity) (Van Straaten and Wensing 1978).

Around PND 25, all populations of perinatal Leydig cells begin to regress; however, involution of the interstitium is more pronounced at PND 35 (Van Straaten and Wensing 1978). Regressed Leydig cells retain their shape, but their cell volume is decreased, largely due to the loss of sER (van Vorstenbosch et al. 1984). Likewise, steroidogenic enzyme abundance also diminishes (Van Straaten and Wensing 1978). Reductions in cell volume were more pronounced in intertubular versus peritubular Leydig cells, suggesting that peritubular Leydig cells do not fully differentiate before regression and may therefore differ functionally in adulthood (Van Straaten and Wensing 1978). In sum, two types of perinatal Leydig cells arise in the pig, with intertubular Leydig cells predominating in the perinatal testis (Van Straaten and Wensing 1978).

As a result of the abundant Leydig cell proliferation between 95 DPC and PND 21, testicular composition changes dramatically during the perinatal period. Testis weight, Leydig cell volume and nuclear volume, total Leydig cell weight, and the percentage of Leydig cells within the testis increase (Van Straaten and Wensing 1978). Accordingly, testosterone secretion also increases (Colenbrander et al. 1977). Testosterone production by perinatal Leydig cells is still relatively low at the end of gestation (<0.5 ng/mL) (Ford et al. 1980) but increases (∼1.5 ng/mL) after birth and remains elevated for approximately 3 wk (Moon and Hardy 1973).

**Figure 2:**
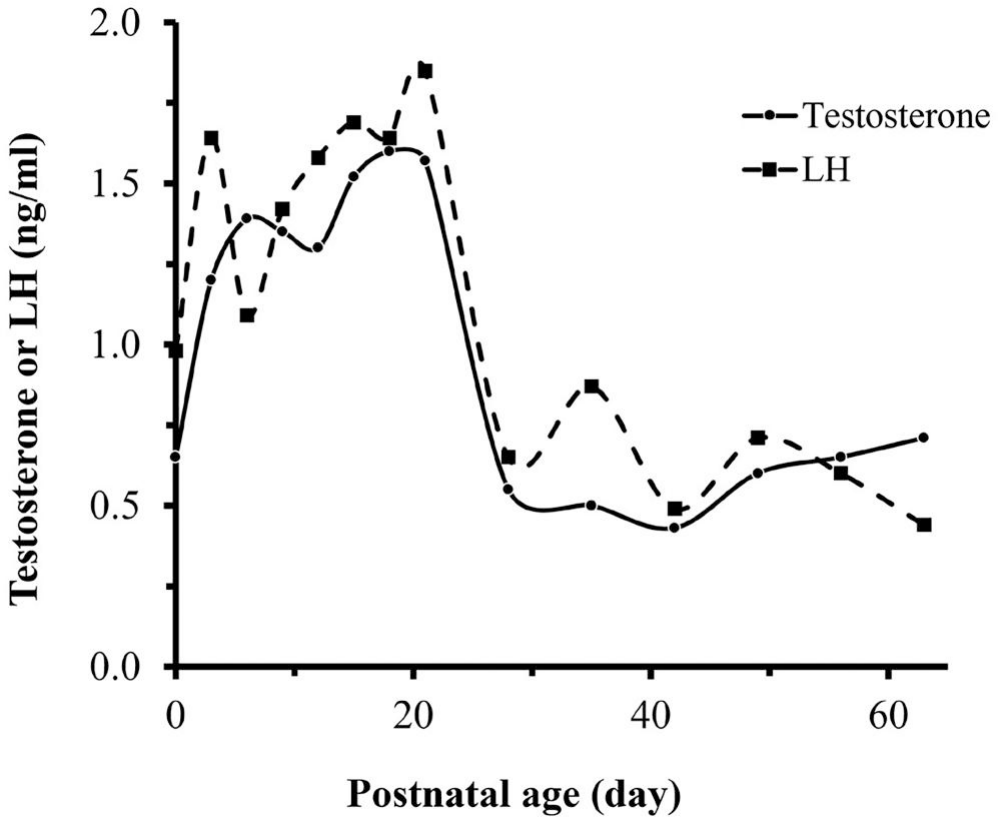
Testosterone and luteinizing hormone production in the neonatal boar. Serum hormone concentrations were measured in boars from birth to approximately 2 mo of age. Data replotted from Ford (1983a) and Ford and Schanbacher (1977).

The advent of cell proliferation and testosterone production in perinatal Leydig cells is likely stimulated by a preceding rise in the production of LH (van Vorstenbosch et al. 1984). Consistent with this, perinatal Leydig cells have high numbers of LH receptors (80,000/cell), even compared with adult Leydig cells (35,000/cell) (Ford and Schanbacher 1977). Interestingly, perinatal Leydig cells do not develop normally in decapitated fetuses, demonstrating a clear dependency on pituitary-derived LH (van Vorstenbosch et al. 1984). In a study by Ford and Schanbacher (1977), treatment with antiserum against LH reduced testosterone production in PND 2 boars. Hence, testosterone production from perinatal Leydig cells is reliant on LH. Thus, both LH and testosterone concentrations are elevated in neonates (Figure 2) (Colenbrander et al. 1977).

Classically, elevated testosterone would feedback on the HPG axis to reduce LH production. However, during the perinatal period, this feedback system is absent, suggesting that these regulatory mechanisms are not yet fully established in the neonatal boar (Moon and Hardy 1973; Ford and Schanbacher 1977). This finding likely reflects reduced sensitivity of the HPG axis to gonadal steroids, allowing both testosterone and LH to remain elevated during the perinatal period (Figure 2) (Colenbrander et al. 1977). Maturation of the HPG axis appears to occur after PND 21 (Ford and Schanbacher 1977). For example, Ford and Schanbacher (1977) demonstrated that castration on PND 3 quickly reduced blood testosterone concentrations without a compensatory rise in LH production. After PND 21, however, LH was greater in the circulation of barrows compared with boars. These results appear to be age-specific (and not due to weaning stress) because weaning did not occur until 6 wk of age in their study (Ford and Schanbacher 1977). The mechanisms behind this phenomenon remain unknown. Ford and Schanbacher (1977) documented that both LH and testosterone decline after PND 21. Moon and Hardy (1973) reported that both LH and testosterone levels decrease after PND 25, an age that coincides with Leydig cell atrophy (Van Straaten and Wensing 1978).

After the regression of perinatal Leydig cells around PND 21, immature Leydig cells within the testis produce low levels of steroid hormones (Van Straaten and Wensing 1978). Production of LH is also low (Colenbrander et al. 1978) and these Leydig cells are hyporesponsive to LH stimulation due to reduced: 1) cell volume and abundance of intracellular steroidogenic organelles, particularly mitochondria (Allrich et al. 1982; Lunstra et al. 1986); 2) activity of steroidogenic enzymes (Van Straaten and Wensing 1978); and 3) expression of LH receptors (Ford and Schanbacher 1977). Thus, the steroid-producing capacity of immature Leydig cells in the pre-pubertal testis is low. However, circulating testosterone levels gradually increase between PND 40 and 100 (Colenbrander et al. 1978) without concomitant increases in LH production. This discrepancy can possibly be explained by an 8-fold increase in the number of immature Leydig cells in the developing pre-pubertal testis (Allrich et al. 1982).

### Adult Leydig cells

The final phase in Leydig cell development starts between 100 and 130 d of age (Van Straaten and Wensing 1978). The stimulus for initiation of this final developmental phase is not clear since LH production does not increase during pubertal attainment (Colenbrander et al. 1978; Allrich et al. 1983). However, LH may still be involved since the number of LH receptors per Leydig cell increases after 100 d of age (Lunstra et al. 1986), indicating increased LH sensitivity. Moreover, peritubular Leydig cells expand (increased cell volume and nuclear size) until reaching a peak cell volume (3,572 µm^3^) by 160 d of age, with the volume declining to approximately 2,000 µm^3^ post-puberty (Lunstra et al. 1986). Additionally, the number of peritubular Leydig cells also increases, although it is unclear if this increase is due to the contribution of Leydig stem cells or mitosis of differentiated Leydig cells (Allrich et al. 1982). Interestingly, peritubular Leydig cells develop in groups; some clusters differentiate fully whereas others remain unchanged. Steroidogenic activity of the peritubular Leydig cells is robust during this developmental window (Van Straaten and Wensing 1978). In contrast, intertubular Leydig cells remain quiescent, with low steroidogenic enzyme activity, no proliferation and a reduction in cell volume. Intertubular Leydig cells also remain separate from peritubular Leydig cells, primarily along the testicular septa (Van Straaten and Wensing 1978). Therefore, the peritubular Leydig cells expand to fill the interstitial compartment and become the terminally differentiated adult Leydig cell population (Van Straaten and Wensing 1978). Although, two types of adult Leydig cells exist, peritubular Leydig cells are the predominant cell type in the adult testis (Van Straaten and Wensing 1978).

Pubertal attainment has many definitions in the male. Herein, the transition between a pre-pubertal and a post-pubertal animal will be referred to as the peripubertal phase (between 100 and 160 d of age). During the peripubertal phase, testicular weight increases 3- to 4-fold (Van Straaten and Wensing 1978; Allrich et al. 1982). During this time, there is also a large increase in the percentage of the testis that is comprised of seminiferous tubules (Allrich et al. 1982), since the diameter of each seminiferous tubule increases 2-fold (Van Straaten and Wensing 1978; Allrich et al. 1982). Further, the number of germ cells in each tubule doubles and spermatozoa become detectable in the tubular lumen (Allrich et al. 1982). In addition, Leydig cell number doubles (Allrich et al. 1982). Given the drastic increase in seminiferous tubule area, however, the percentage of the testis that is comprised of Leydig cells actually decreases (Allrich et al. 1982). However, the total number of Leydig cells within the testis still rises during this period due to a marked increase in testicular volume (Allrich et al. 1982). Accordingly, production of testosterone, 17β-estradiol and estrone sulfate all increase by approximately 2-fold during this time period (Colenbrander et al. 1978).

**Figure 3:**
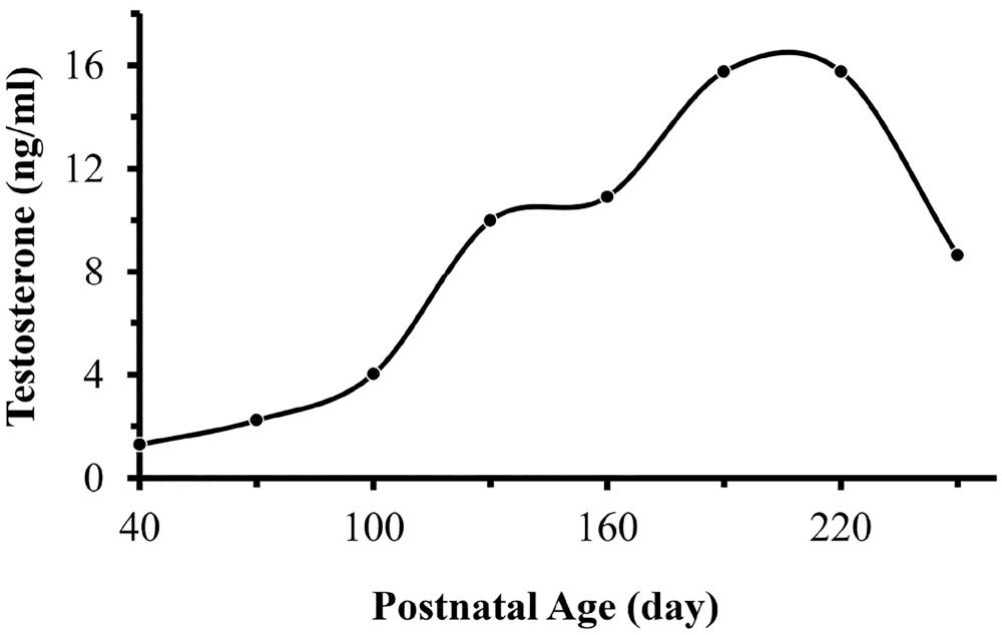
Testosterone production in the maturing boar. Serum testosterone concentrations were measured in boars from postnatal d 40 to 250. Data replotted from Allrich et al. (1982).

**Figure 4:**
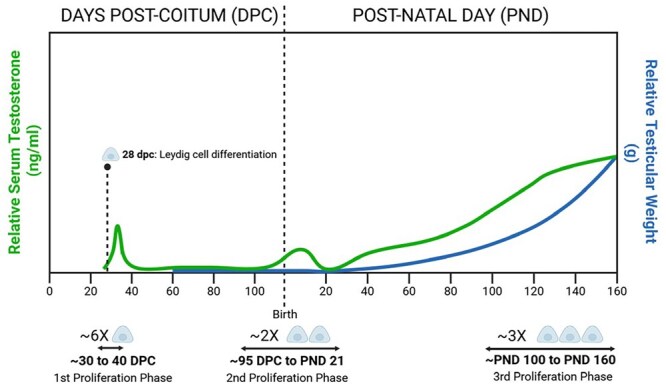
Leydig cell development and function across the lifespan in boars. Leydig cells begin to differentiate ∼28 d post-coitus (DPC) in swine. Testosterone production from fetal Leydig cells peaks ∼35 DPC. Testosterone production is transiently elevated during perinatal life. Testosterone production steadily rises during pubertal development. Leydig cell proliferation occurs during fetal (6-fold), perinatal (2-fold), and pre-pubertal (3-fold) life. Testis weight steadily increases during pubertal development.

During the peripubertal period, Leydig cell steroidogenic output peaks and then declines in adulthood (Figure 3) (Colenbrander et al. 1978). This high steroid-producing capacity coincides with transient increases in Leydig cell volume and steroidogenic organelles, which also decrease by adulthood (Lunstra et al. 1986). However, LH secretion (Colenbrander et al. 1978) and the number of LH receptors per Leydig cell are similar between peripubertal (130–160 d of age) and pubertal (220–250 d of age) animals (Allrich et al. 1982). Thus, the mechanism that reduces testosterone production by adulthood is unclear. Nevertheless, these data suggest that peaks in androgens are necessary for pubertal development of the boar. Allrich et al. (1982) speculated that especially high levels of testosterone or its metabolites may be needed during the peripubertal period in order to prime reproductive behaviors, initiate spermatogenesis and/or stimulate accessory sex gland function. A summary of Leydig cell developmental phases is available in Figure 4.

## Leydig cell ultrastructure in the pig

**Figure 5:**
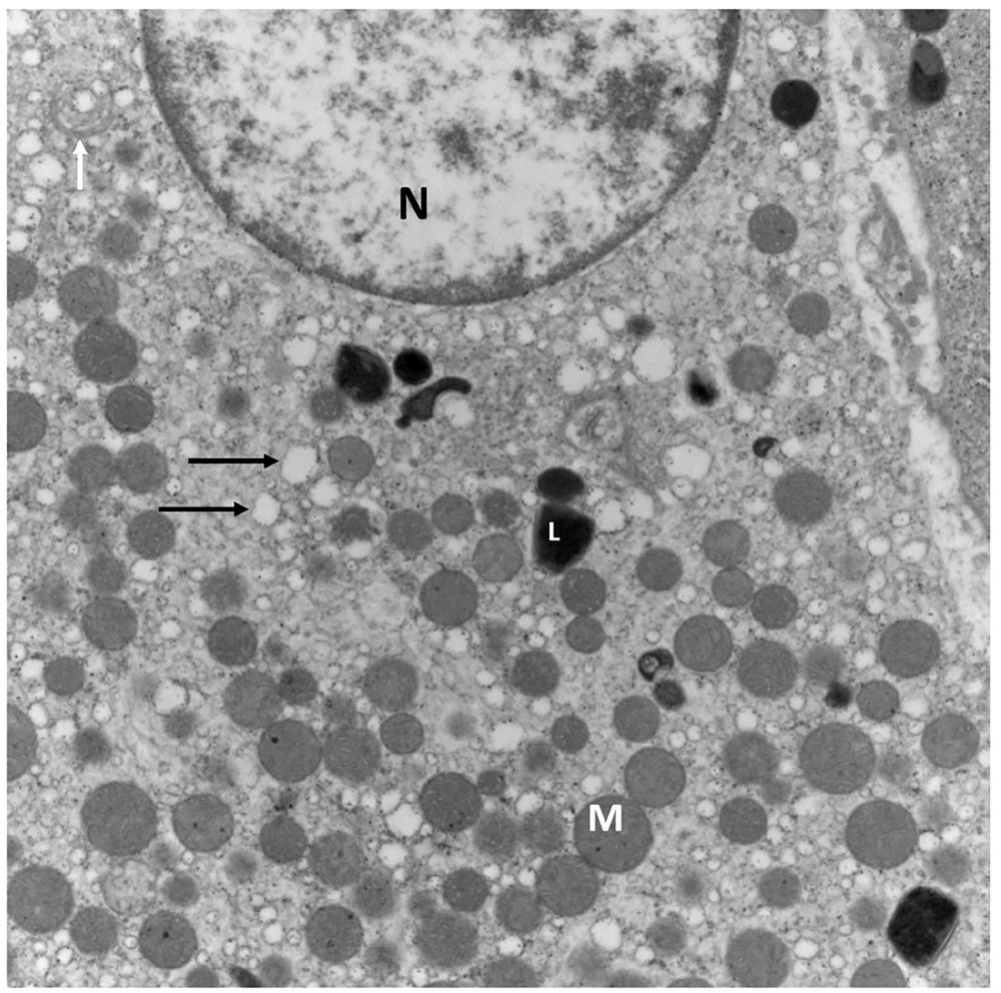
High-magnification electron micrograph of a Leydig cell from an adult white crossbred boar (×18,600). A portion of the nucleus (N) is visible, along with abundant spherical mitochondria (M) and extensive, vesiculated smooth endoplasmic reticulum (black arrow), characteristic of steroidogenic cells. Lysosomes (L) and a Golgi apparatus (white arrow) are also visible.

Several reports have detailed the ultrastructure of the porcine Leydig cell (Pelliniemi 1975; Van Straaten and Wensing 1978; van Vorstenbosch et al. 1982, 1984; Aguas 1983; Ford 1983a; Lunstra et al. 1986). Porcine Leydig cells appear polygonal to ovoid in shape with a large, eccentrically positioned nucleus containing one or more nucleoli (van Vorstenbosch et al. 1982) and contain abundant steroidogenic organelles (Lunstra et al. 1986). Electron microscopy revealed numerous mitochondria, often round or rod-like and concentrated near the nucleus, as well as extensive sER, which occupies approximately 68% of the cytoplasm (Lunstra et al. 1986). Rough endoplasmic reticulum is sparse, and lipid droplets containing esterified cholesterol are present (van Vorstenbosch et al. 1982; Lunstra et al. 1986). A representative electron micrograph of an adult porcine Leydig cell is shown in Figure 5. Detailed quantification of porcine Leydig cell ultrastructure from adult boars was performed by Lunstra et al. (1986) and these results are summarized in Table 1.

**Table 1:** Quantification of Leydig cell ultrastructure in adult boars.^a^

Organelle	Volume^b^	Percentage^c^
**Nucleus**	303	14
**Total cytoplasm**	1,875	86
**Smooth endoplasmic reticulum and Golgi apparatus**	1,271	58
**Mitochondria**	322	15
**Granular vesicles**	45	2
**Multivesicular bodies**	122	6
**Lysosomes**	46	2
**Lipid droplets**	25	1
**Rough endoplasmic reticulum**	44	2

a Adapted from Lunstra et al. (1986).

b Volume (μm^3^/Leydig cell).

c Percentage of total Leydig cell volume.

## Testicular steroidogenesis in pigs

Steroidogenesis predominantly occurs within the adrenal gland, gonads, and placenta (in females). Unlike protein hormones, few steroid hormones are stored within the cell (Russell 1996). Thus, synthesis of steroid hormones must occur de novo. Secretion of steroid hormones is regulated in three ways: 1) increased delivery of cholesterol to the mitochondria (minutes); 2) increased steroidogenic gene expression (hours to days); and 3) stimulation of steroidogenic cell proliferation (days to months) (Russell 1996). The first mechanism is considered the acute steroidogenic response whereas the latter two demonstrate chronic steroidogenic responses. Together, these mechanisms govern the production of steroid hormones (Russell 1996).

The primary function of Leydig cells within the testis is the production of sex steroids. The pig testis is unique among livestock species with the largest interstitial compartment and highest abundance of Leydig cells, suggesting a high capacity for steroid production (Miller 2013). Indeed, numerous sex steroids have been detected in venous testicular blood of boars (Table 2) as well as in circulation (Table 3). Androgens, estrogens, and progestogens detected in circulation are predominantly produced by the testis; the adrenal gland contributes minimally to circulating concentrations of these sex steroids (Table 4).

**Table 2:** Steroid hormones detected in testicular vein serum of the boar.^a^

C^19^	
	Testosterone
	Androstenedione
	Dehydroepiandrosterone
	Dehydroepiandrosterone sulfate
	Epiandrosterone sulphate
	Androstenediol sulphate
	5α-Androstene-3β, 17β-diol sulphate
	11β-Hydroxytestosterone
	11β-Hydroxyandrostenedione
	19-Hydroxytestosterone
	19-Hydroxyandrostenedione
	5α-Androst-16-en-3α-ol sulphate
	5α-Androst-16-en-3β-ol sulphate
	5α-Androst-16-en-3-one
	5,16-Androstadien-3β-ol sulphate
**C^18^**	
	19-Nortestosterone
	Estradiol-17β sulphate
	Estrone sulphate

a Adapted from Raeside et al. (2006). Steroids are grouped by carbon number (C^19^ and C^18^) to reflect their biosynthetic classification.

**Table 3:** Circulating concentrations of gonadal steroid hormones in adult boars.^a^

Hormone	Concentration		References^b^
	ng/mL	nM	
**Pregnenolone**	6.0	19.0	Wichmann et al. (1984)
**Progesterone**	0.03	0.09	Desaulniers (2018)
**17α*-*Hydroxyprogesterone**	0.08	0.25	Desaulniers (2018)
**Dehydroepiandrosterone (DHEA)**	0.30	1.04	Desaulniers (2018)
**DHEA sulfate**	49.7	134.9	Desaulniers (2018)
**Androstenedione**	0.28	0.98	Desaulniers (2018)
**Testosterone**	1.56	5.4	Desaulniers (2018)
**Dihydrotestosterone**	0.15	0.54	Desaulniers (2018)
**Androsterone**	0.41	1.42	Desaulniers (2018)
**Androstenediol**	0.55	1.9	Wichmann et al. (1984)
**Estrone**	0.11	0.37	Desaulniers (2018)
**17β-Estradiol**	0.10	0.38	Desaulniers (2018)

a Values are adapted from the cited sources. Nanomolar (nM) and mass (ng/mL) concentrations were interconverted using molecular weight.

b Values were derived from studies using either venipuncture (Wichmann et al. 1984) (*n *= 10; Oldenburger Landedelschwein boars) or jugular cannula (Desaulniers 2018) (*n *= 5; white crossbreed boars). Jugular cannulation minimizes handling stress and may better reflect basal circulating concentrations.

**Table 4:** Relative contribution of the adrenal gland and testis to circulating progestogens, androgens, and estrogens in boars.^a^

Hormone	Adrenal gland^b^	Testis
**17α*-*Hydroxyprogesterone,%**	2.6	97.4
**Progesterone, %**	3.7	96.3
**Dehydroepiandrosterone, %**	1.7	98.3
**Dehydroepiandrosterone sulphate, %**	28.9	71.1
**Androstenedione, %**	2.2	97.8
**Testosterone, %**	1.7	98.3
**Dihydrotestosterone, %**	4.6	95.4
**Androsterone, %**	3.0	97.0
**Estrone, %**	3.7	96.3
**17β-estradiol, %**	2.6	97.4

a Derived by comparing serum concentrations of steroid hormones in adult white crossbred boars (*n* = 2) prior to and 20 d after castration. Samples were collected under basal conditions via jugular cannulae.

b Values may overestimate the proportion of steroid hormones originating from the adrenal gland, as calculations assume that adrenal steroid production does not increase following castration.

**Figure 6:**
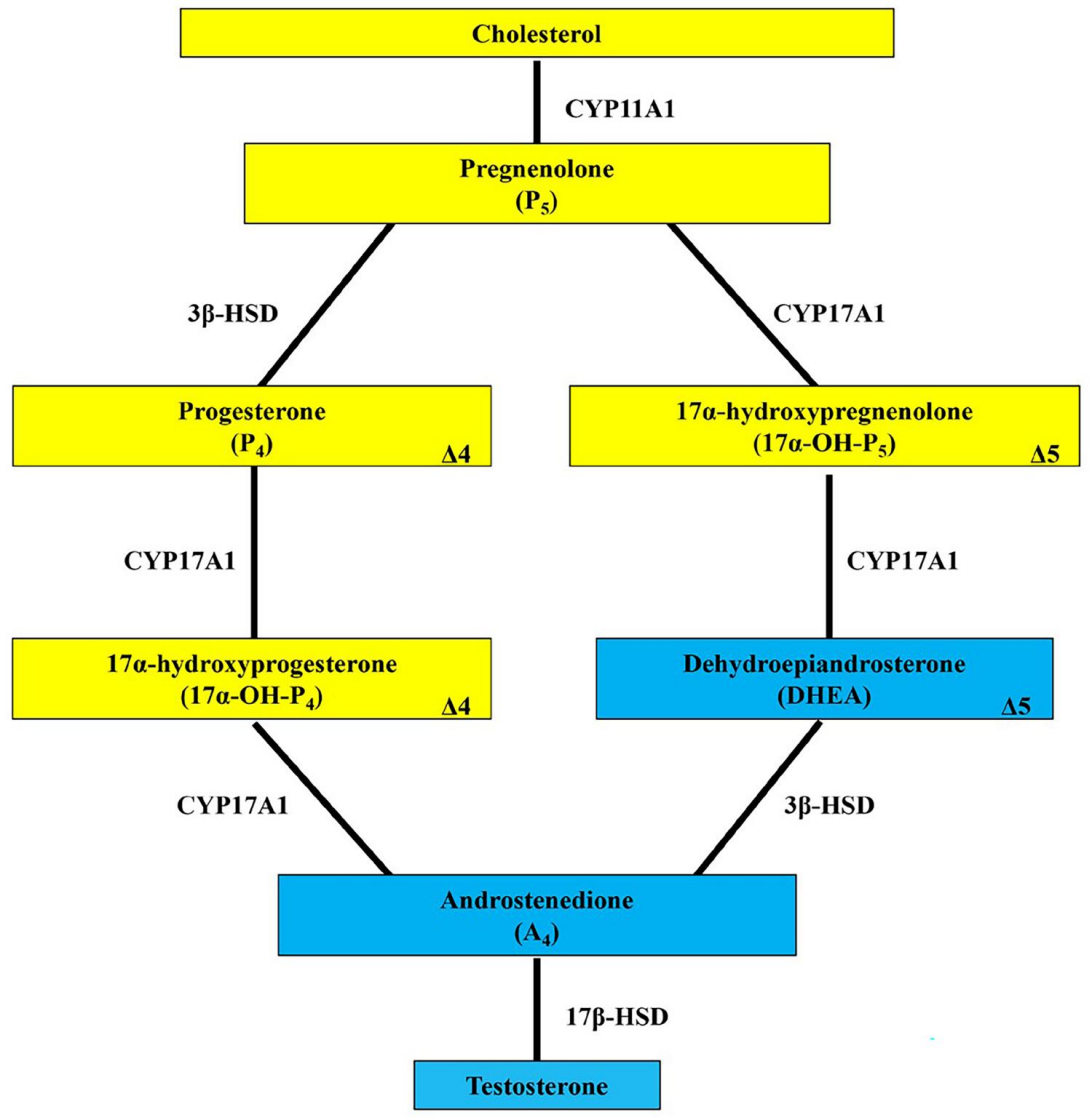
The Δ4 and Δ5 pathways of testosterone production in the testis. Boars predominately use the Δ5 pathway to produce testosterone. Enzymes: Cytochrome P450 family 11 subfamily A member 1 (CYP11A), cytochrome P450 family 17 subfamily A member 1 (CYP17A), 3β-hydroxysteroid dehydrogenase (3β-HSD), and 17β-hydroxysteroid dehydrogenase (17β-HSD). Steroid products: Pregnenolone (P_5_), Progesterone (P_4_), 17α-hydroxyprogesterone (17OH-P_4_), 17α-hydroxypregnenolone (17OH-P_5_), dehydroepiandrosterone (DHEA), androstenedione (A_4_), and testosterone. Yellow boxes denote progestogens and blue boxes denote androgens. Adapted from Conley and Bird (1997) and Häggström and Richfield (2014).

The most prominent steroid hormone produced by the testis of boars is testosterone (Table 5) (Fawcett et al. 1973). Testosterone biosynthesis can occur via two steroidogenic pathways, Δ4 or Δ5 (Figure 6). These names originated because the steroid intermediates retain a double bond at either carbon 4–5 (Δ4) or carbon 5–6 (Δ5) (Conley and Bird 1997). The specific pathway utilized (Δ4 or Δ5) is determined based on enzymatic competition between CYP17A1 and 3β-HSD for pregnenolone, as well as availability of Δ4/Δ5 intermediates (Raeside et al. 2006). Humans, non-human primates, sheep, and cattle predominantly utilize the Δ5 pathway, whereas mice, rats, and pigs can use either the Δ4 or Δ5 pathway (Raeside et al. 2006). However, in the boar testis, testosterone production primarily occurs via the Δ5 route of steroidogenesis (Figure 6) (Conley 1992).

**Table 5:** Serum steroid hormone concentrations in the adult boar relative to testosterone (%).^a^

Hormone	Relative to testosterone, %^b^	References^c^
**Pregnenolone**	111.7	Wichmann et al. (1984)
**Progesterone**	1.7	Desaulniers (2018)
**17α*-*Hydroxyprogesterone**	4.6	Desaulniers (2018)
**Dehydroepiandrosterone (DHEA)**	19.3	Desaulniers (2018)
**DHEA sulfate**	2498.1	Desaulniers (2018)
**Androstenedione**	18.1	Desaulniers (2018)
**Dihydrotestosterone**	10.0	Desaulniers (2018)
**Androsterone**	26.3	Desaulniers (2018)
**Androstenediol**	10.3	Wichmann et al. (1984)
**Estrone**	6.9	Wichmann et al. (1984)
**17β-Estradiol**	7.0	Desaulniers (2018)

a Values are adapted from the cited sources. Nanomolar (nM) and mass (ng/mL) concentrations were interconverted using molecular weight.

b Values were calculated relative to testosterone concentrations measured within the same study (testosterone = 100%), as reported in the original source.

c Values were derived from studies using either venipuncture (Wichmann et al. 1984) (*n *= 10; Oldenburger Landedelschwein boars) or jugular cannula (Desaulniers 2018) (*n *= 5; white crossbreed boars). Jugular cannulation minimizes handling stress and may better reflect basal circulating concentrations.

## Cholesterol availability

Unesterified (free) cholesterol is the precursor for all steroid hormones; therefore, it must be readily available to support acute steroidogenesis (Mutembei et al. 2005). Cholesterol within steroidogenic cells is derived from three sources: 1) extracellular import; 2) intracellular reserves; or 3) de novo synthesis (LaVoie 2017). Circulating lipoproteins are the primary source of cholesterol for steroidogenesis (Mutembei et al. 2005). Pigs utilize both low density lipoproteins (LDL) and high density lipoproteins (HDL) for steroidogenesis in the ovary (Rajkumar et al. 1989); however, in the porcine testis, LDL is the major substrate for steroidogenesis (Benahmed et al. 1981, 1983). LDL bind to LDL receptors on Leydig cells and undergo endocytosis. After internalization, cholesterol is removed from lipoproteins in lysosomes.

Free (unesterified) cholesterol can be utilized immediately for steroidogenesis or esterified for storage in intracellular lipid droplets as cholesteryl esters (Russell 1996; LaVoie 2017). However, the ester group must first be removed by hormone-sensitive lipase to yield bioavailable cholesterol (Russell 1996; LaVoie 2017). The final source of cholesterol for steroidogenesis is produced de novo in the sER from acetate. However, this mechanism requires increased energy and is only utilized when other sources are unavailable (Mutembei et al. 2005). Indeed, Benahmed et al. (1983) demonstrated that only 25% of maximal steroidogenic capacity is from de novo synthesis of cholesterol in porcine Leydig cells. Once unesterified cholesterol becomes available, it must be transported to the mitochondria to enter the steroidogenic pathway (Mutembei et al. 2005). It is commonly believed that StAR transports cholesterol to the mitochondria, but this is a misnomer. StAR resides on the outer surface of the mitochondria and is responsible for the transport of cholesterol from the outer to the inner mitochondrial membrane; transport to the mitochondria from the cytosol appears to involve other mediators (Mutembei et al. 2005).

## Steroidogenic proteins

### Steroid acute regulatory protein

Steroid acute regulatory protein is a critical regulator of acute steroidogenesis (Stocco 1997). StAR is produced as a 37 kDa precursor protein with a lipid-binding domain and an N-terminus mitochondrial targeting signal (Russell 1996). This phosphoprotein resides on the outer mitochondrial membrane where it binds free cholesterol; this step is dependent upon phosphorylation at Ser195, which promotes the necessary conformational change to bind cholesterol (Arakane et al. 1997). After binding, StAR translocates one molecule of cholesterol from the outer to the inner mitochondrial membrane. Little is known about StAR in the pig, although it is assumed to function similarly to other species (Robic et al. 2014). The porcine StAR gene is 87.3% homologous to the human protein (Robic et al. 2014). Interestingly, StAR was highly expressed in testicular tissue of boars with high levels of androstenone in their fat, a steroid hormone associated with boar taint (Leung et al. 2010). Expression of StAR is primarily mediated by the activation of protein kinase A (PKA) and cyclic adenosine monophosphate (cAMP) after stimulation by LH (Russell 1996).

### Cytochrome P450 family 11 subfamily A member 1

Cholesterol side-chain cleavage enzyme (P450scc), or CYP11A1, is the first enzyme in the steroidogenic pathway. In the testis, CYP11A1 solely localizes to the Leydig cell (Payne and Hales 2004). Within the cell, CYP11A1 is membrane bound in the inner mitochondrial membrane (Russell 1996). The ultimate role of CYP11A1 is to cleave the side-chain of cholesterol during steroidogenesis to produce pregnenolone (Payne and Hales 2004). Transcriptional regulation of CYP11A1 is governed by trophic hormones. In the testis, that hormone is LH, which regulates CYP11A1 levels via PKA-dependent signaling (Hanukoglu 2006). Accordingly, treatment with cAMP increases expression of *CYP11A1* whereas LH-deprivation quickly reduces *CYP11A1* mRNA levels (Gomez et al. 2013).

### Cytochrome P450 family 17 subfamily A member 1

CYP17A1 is membrane bound in the sER of Leydig cells and is responsible for the generation of both 17α-hydroxypregnenolone and dehydroepiandrosterone (DHEA) (Payne and Hales 2004). First, CYP17A1 converts pregnenolone to 17α-hydroxypregnenolone by 17α-hydroxylation. Then, the C17-C20 carbon bond of 17α-hydroxypregnenolone is cleaved, yielding DHEA. Therefore, CYP17A1 confers both 17α-hydroxylase and 17,20-lyase activity, depending on the substrate (Garcia et al. 2012). Expression of CYP17A1 is thought to be regulated by LH in a dose- and time-dependent manner due to the activity of cAMP (Gomez et al. 2013). However, others have demonstrated that lower concentrations of cAMP exert a greater stimulatory effect on transcription and translation than higher concentrations, which may be due to inhibitory effects of increased testosterone secretion. Indeed, in conditions of high cAMP levels, mRNA production was further enhanced by use of an inhibitor of cholesterol biosynthesis (aminoglutethimide) (Zhang et al. 1995). Thus, CYP17A1 is cAMP-dependent and is inhibited by testosterone stimulation.

### 3β-Hydroxysteroid dehydrogenase

Within the pig testis, 3β-HSD is expressed solely in Leydig cells (Payne and Hales 2004). In many species, 3β-HSD is present in both the mitochondria and sER (Payne and Hales 2004). However, in pigs, 3β-HSD appears to be solely present in the sER (Cooke 1991). Interestingly, there are numerous genes and resulting isozymes of 3β-HSD in other species (Payne and Hales 2004). However, the presence of 3β-HSD isozymes in pigs is unclear (Robic et al. 2014). Sequencing data suggest that pigs only have one 3β-HSD gene, which catalyzes the conversion of DHEA to androstenedione (Robic et al. 2014). In other species, 3β-HSD is LH/cAMP responsive (Keeney and Mason 1992a, 1992b), but the regulatory mechanisms in the boar testis remain poorly defined (Rasmussen et al. 2013).

### 17β-Hydroxysteroid dehydrogenase

17β-HSD is a membrane-bound enzyme present throughout the sER of Leydig cells (Ohno et al. 2005). In many species, isozymes of 17β-HSD exist, all with different tissue/cell specificities and kinetics (Hanukoglu 1992). Data suggests that the pig testis maintains only one type, 17β-HSD type 3 (Hanukoglu 1992; Robic et al. 2014); it was first isolated from microsomal fractions of porcine testicular samples in the early 1970s (Ohno et al. 2006). 17β-HSD type 3 catalyzes the conversion of androstenedione to testosterone (Ohno et al. 2006). Like 3β-HSD, 17β-HSD expression is regulated by LH in the testis through the activation of the PKA pathway. Thus, expression of 17β-HSD can be regulated by modulating cAMP levels within the Leydig cell (Inano and Tamaoki 1974). Interestingly, a synthetic PKC analogue also increases 17β-HSD expression; an effect which becomes synergistic upon the addition of a cAMP analogue. Therefore, these data suggest that 17β-HSD is partially regulated in a non-classical, PKA-independent manner (Inano and Tamaoki 1974).

### Cytochrome P450 family 19

Contrary to many other species, pigs produce three isozymes of CYP19, which is also known as aromatase (Shi et al. 2007). Thus, the CYP19 gene was triplicated in pigs (Shi et al. 2007). However, isozyme expression appears to be tissue-specific. Three different isozymes have been separately identified in the porcine placenta, embryo, and gonads (Conley et al. 2009). In pigs, the testis-specific isozyme (CYP19A3) is present within the sER of Leydig cells (Conley et al. 1997; Corbin et al. 1999, 2009; Ziegler et al. 1999; Fraczek et al. 2001; Vanselow et al. 2021). The substrate for CYP19 is testosterone, whereas the product is 17β-estradiol (Conley et al. 2009).

Bourguiba et al. (2003) demonstrated that CYP19 expression in Leydig cells is regulated by several hormones. For example, expression was upregulated by testosterone and the gonadotropins, both of which enhanced estrogen output (Bourguiba et al. 2003). Similarly, dihydrotestosterone (DHT) treatment enhanced expression of CYP19, without altered estrogen output as DHT is not aromatizable. Further, CYP19 expression is also upregulated with exposure to Sertoli cell-derived factors (Bourguiba et al. 2003). In contrast, estrogen treatment downregulates CYP19 transcription (Bourguiba et al. 2003). Activity of CYP19 can be blocked by a CYP19 inhibitor, such as letrozole (At-Taras et al. 2006). Letrozole is highly selective for CYP19 and works by competing with testosterone for binding (Bhatnagar 2007). Functionally, conversion of testosterone into estrogen is inhibited, which ultimately reduces estrogen production and increases testosterone accumulation (At-Taras et al. 2006).

### Regulation of steroidogenesis via luteinizing hormone

Steroidogenesis within Leydig cells is classically regulated via the HPG axis. Luteinizing hormone synthesized and released from the anterior pituitary gland binds to its receptor on the plasma membrane of Leydig cells, causing a conformational change that activates the G-protein, G_s_. Then, G_s_ activates adenylate cyclase, which stimulates the production of cAMP from adenosine triphosphate (ATP). Next, cAMP binds to the regulatory subunits of PKA stimulating dissociation of the catalytic subunits. The catalytic subunits of PKA phosphorylate existing steroidogenic proteins within the cell, such as StAR. Protein kinase A also activates cAMP response element-binding protein (CREB), stimulating the expression of genes encoding StAR as well as the steroidogenic enzymes (CYP11A1, CYP17A1, 3β-HSD, 17β-HSD, and CYP19) within the Δ5 pathway (Gower 1972).

**Figure 7:**
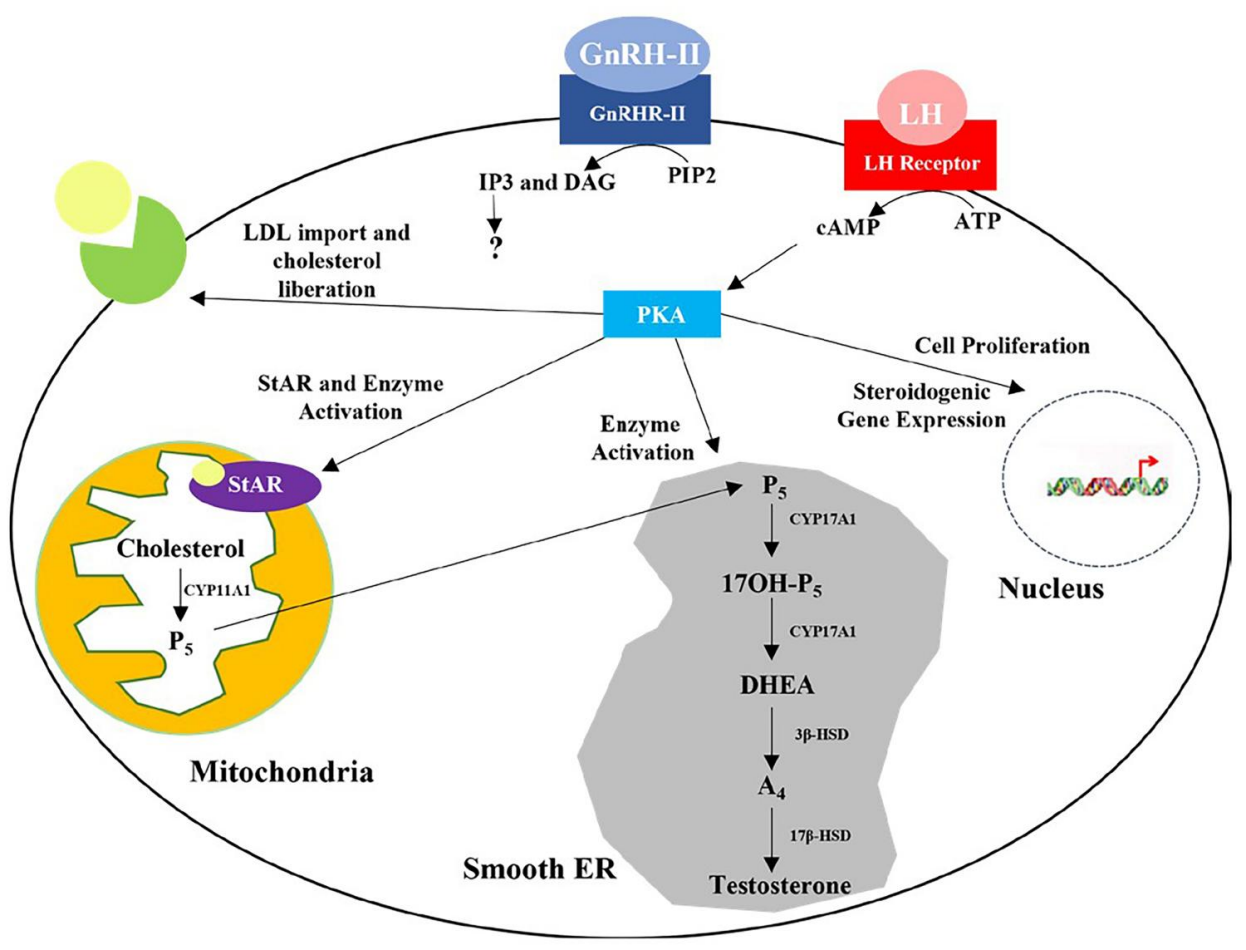
The steroidogenic pathway within porcine Leydig cells. Luteinizing hormone (LH) binding to the LH receptor activates the G_s_ protein–adenylyl cyclase–cAMP signaling cascade, leading to activation of protein kinase A (PKA). PKA stimulates steroidogenic acute regulatory protein (StAR), promoting cholesterol transport to the inner mitochondrial membrane. In the mitochondrion, cholesterol is converted to pregnenolone (P5) by cytochrome P450 side-chain cleavage enzyme (CYP11A1). Pregnenolone enters the smooth endoplasmic reticulum where sequential conversion to 17-hydroxypregnenolone (17OH-P5) and dehydroepiandrosterone (DHEA) occurs via CYP17A1, followed by conversion to androstenedione (A4) via 3β-hydroxysteroid dehydrogenase (3β-HSD) and subsequently to testosterone via 17β-hydroxysteroid dehydrogenase (17β-HSD). LH-dependent signaling also upregulates transcription of steroidogenic genes, further enhancing testosterone synthesis. Gonadotropin-releasing hormone II (GnRH-II) and its receptor (GnRHR-II) mediate steroidogenesis in porcine Leydig cells via unknown mechanisms. GnRHR-II signaling via G_αq/11_ is putative based on the work of Neill et al. (2002a).

In addition to the regulation of gene expression, PKA also phosphorylates StAR to promote cholesterol bioavailability (Russell 1996). Phosphorylated StAR initiates steroidogenesis by delivering cholesterol from the outer to the inner mitochondrial membrane, which is considered the rate-limiting step of steroidogenesis. Next, CYP11A1 converts cholesterol into pregnenolone (P5) (Presicce et al. 2015). Pregnenolone exits the mitochondria via diffusion and enters the sER where it is oxidized to 17α-hydroxypregnenolone (17OH-P5) by the hydroxylase activity of CYP17A1. 17OH-P5 is then further cleaved to DHEA by the lyase activity of CYP17A1. Dehydroepiandrosterone is dehydrogenated via 3β-HSD for conversion into androstenedione, which is cleaved to testosterone by 17β-HSD (Raeside et al. 2006). Unlike many species, pigs express CYP19 within the Leydig cell (Ziegler et al. 1999); therefore, testosterone is converted to 17β-estradiol within Leydig cells. A summary of steroidogenesis within porcine Leydig cells is presented in Figure 7.

### Regulation of steroidogenesis via gonadotropin-releasing hormone II

In addition to LH, Leydig cell steroidogenesis in swine can also be regulated directly via a locally produced decapeptide, gonadotropin-releasing hormone II (GnRH-II), and its specific receptor (GnRHR-II) (Desaulniers et al. 2015, 2017a; Lents et al. 2017; White et al. 2023). Swine are unique in that they are the only domestic livestock species that produce functional GnRH-II and GnRHR-II proteins (Stewart et al. 2009; Desaulniers et al. 2017a; Morgan and Millar 2024). Genes encoding either GnRH-II and/or its receptor are disrupted in other species (Stewart et al. 2009; Desaulniers et al. 2017a; Morgan and Millar 2024). A seminal study published by Zanella et al. (2000) indicated the presence of a functional GnRH receptor in the testis of swine that directly mediated testosterone production *ex vivo*; however, the classical GnRH receptor (GnRHR-I) was undetectable. Interestingly, GnRHR-II was discovered in mammals the following year (Millar et al. 2001; Neill et al. 2001) and was later identified in swine (Neill et al. 2002a, 2002b).

In pigs, both GnRH-II and its receptor are abundantly produced within the testis, suggesting autocrine/paracrine interaction (Desaulniers et al. 2015). Prior studies from our group demonstrated that GnRHR-II is expressed on the plasma membrane of Leydig cells in the adult boar testis where GnRH-II is primarily produced within the seminiferous tubules (Desaulniers et al. 2015). Treatment of boar testicular explant cultures with GnRH-II elicited testosterone production similar to treatment with human chorionic gonadotropin (agonist of LH) (Desaulniers et al. 2015). Moreover, GnRH-II stimulated testosterone production robustly in vivo, independent of LH (Desaulniers et al. 2015). Further, intratesticular injections of GnRH-II stimulated an increase in circulating testosterone concentrations without stimulating LH secretion (Lents et al. 2017).

Subsequently, our group produced a transgenic swine line with reduced endogenous GnRHR-II expression (GnRHR-II KD) to further study the role of this ligand-receptor complex in swine reproduction (Desaulniers et al. 2017b). Notably, GnRHR-II KD boars had reduced testosterone production during pubertal development, but LH was unaffected (Desaulniers et al. 2017b). Circulating concentrations of 17β-estradiol were also reduced in GnRHR-II KD boars during pubertal development compared with littermate controls (White et al. 2023). Together, these data indicate that GnRH-II and its receptor locally regulate steroidogenesis within porcine Leydig cells.

## Role of gonadal steroids

### Androgens in the fetal boar

The surge in testosterone production by fetal Leydig cells early in gestation is responsible for the differentiation of the male reproductive tract and genitalia. Indeed, fetal testosterone levels are greatest just before or during the appearance of sexually dimorphic external genitalia (Ford et al. 1980). Enlargement of the genital tubercle to form the penis occurs by 38 DPC, maximal enlargement of the mesonephroi occurs by 40 DPC and male tract structures are detectable by 55 DPC (Pattern 1948; Marrable 1971). This period of elevated testosterone production, coined the “masculinization programming window,” is critical for proper male reproductive development. Suppression of fetal androgen production during the highly conserved masculinization programming window reduces fertility in mammals (reviewed by Sharpe 2020).

In the pig, early surges in testosterone production from fetal Leydig cells do not appear to program male sexual behavior (Ford and D’Occhio 1989) as in other species (Wilhelm and Koopman 2006). However, androgens (secreted from either fetal or perinatal Leydig cells), via conversion to 17β-estradiol, may masculinize the male neuroendocrine system (Elsaesser and Parvizi 1979; Ford and D’Occhio 1989). For example, LH production was unaffected by treatment of newborn male castrates with estrogen, unlike newborn gilts which responded by reducing LH production (Elsaesser and Parvizi 1979). Moreover, circulating LH concentrations in female pigs treated prenatally with testosterone were less responsive to estrogen-induced LH secretion in adulthood (Elsaesser and Parvizi 1979). These effects were only observed in gilts treated earlier (d 30, 50, 70) versus later (d 90, 106) in gestation (Elsaesser and Parvizi 1979), suggesting that the window for defeminization of the hypothalamic surge center is prior to 90 DPC (Ford and D’Occhio 1989).

Gubernacular development and testis descent occur prenatally in the pig (Heyns and Pape 1991). Transabdominal testicular descent is thought to be mediated by INSL3 from fetal Leydig cells (Vernunft et al. 2016), whereas androgens have been implicated in mediating transscrotal testis descent between 85–90 DPC (Heyns and Pape 1991). However, the production of testosterone is low in male fetuses during this time period (Ford et al. 1980), suggesting that high androgen levels are not required for transscrotal testicular descent (Van Straaten and Wensing 1978). Low levels of androgens must still be important though as inguinoscrotal testis descent was inhibited in fetal piglets treated with an androgen receptor antagonist (flutamide) before 74 DPC; flutamide treatment also prevented gubernacular regression (McMahon et al. 1995; Barthold et al. 1996). Indeed, the androgen receptor has been detected in the fetal pig gubernaculum (Heyns and Pape 1991). Therefore, the current data suggest that gubernacular outgrowth in the fetal pig occurs during a period of low testosterone production and gubernacular regression occurs when circulating androgen concentrations are increasing (Heyns and Pape 1991).

**Figure 8:**
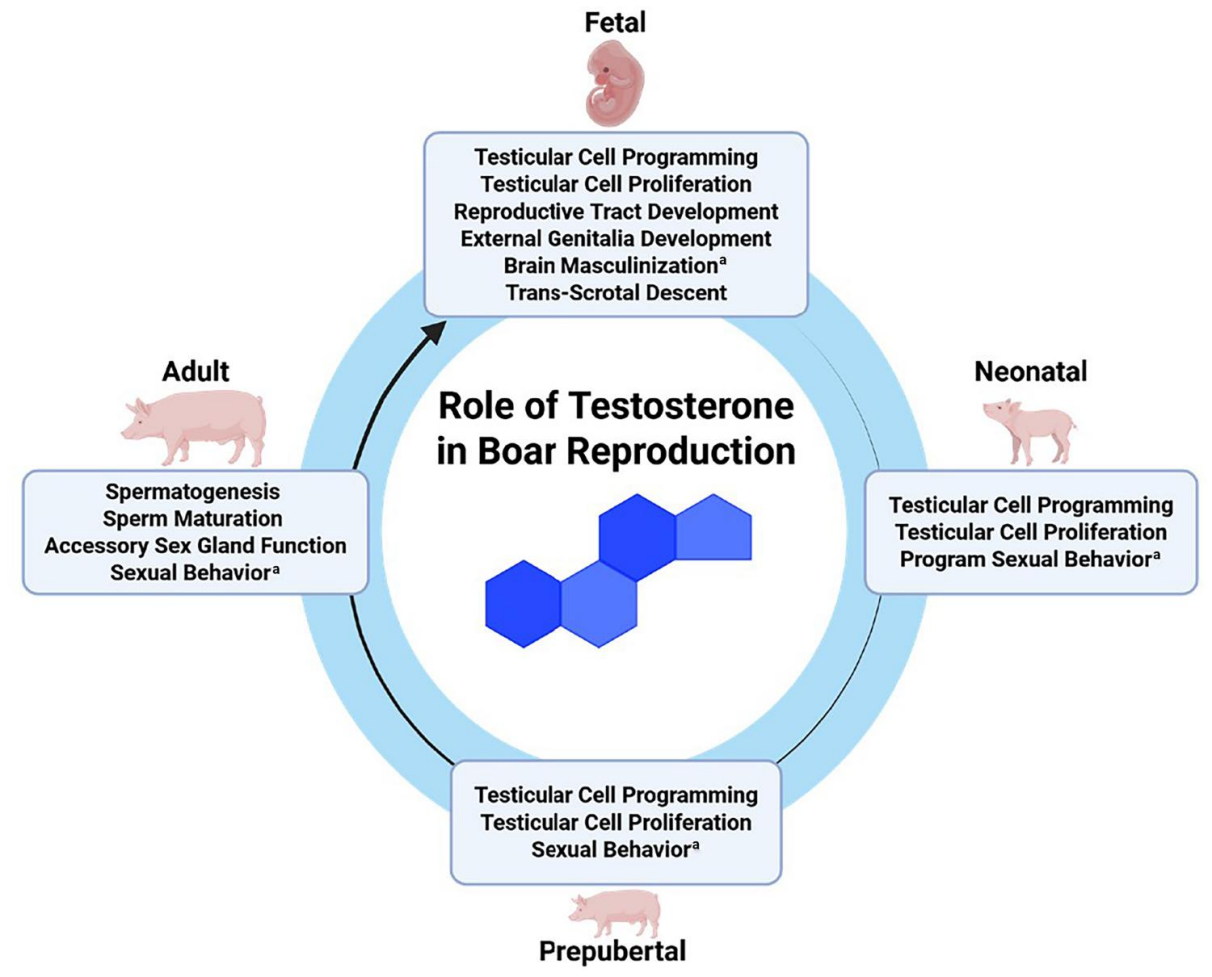
Roles of testosterone in boar reproduction across the lifespan. Processes are grouped by the developmental window. Superscript “a” denotes actions mediated through the aromatization of testosterone to 17β-estradiol.

Androgen production from fetal testes during gestation is also important for the development and function of adult Leydig cells. For example, boars treated with flutamide prenatally (20–28 DPC or 80–88 DPC) had reduced Leydig cell numbers and LH receptor gene expression in adulthood (Kotula-Balak et al. 2012). Flutamide-treated boars also produced reduced serum testosterone concentrations. In contrast, serum 17β-estradiol levels were greater (80–88 DPC only) due to increased production of CYP19 (Kotula-Balak et al. 2012), despite normal secretion of LH and FSH (Kotula-Balak et al. 2012). Thus, these data suggest that fetal androgens are important regulators of Leydig stem cells (Kotula-Balak et al. 2012). Likewise, fetal androgens also affect developing Sertoli cells. For example, disruption of fetal androgen production reduced the expression of tight junction proteins in the adult boar testis, which are critical for integrity of the blood-testis barrier (Kopera et al. 2011; Hejmej et al. 2012). In addition, proliferation of Sertoli cells and pre-spermatogonia has been linked to fetal androgen synthesis (Atanassova et al. 2005; Tan et al. 2005; Zhou et al. 2005; Merlet et al. 2007). Thus, fetal androgens have several diverse effects on male reproductive development (Figure 8).

### Androgens in the neonatal boar

Testosterone plays an important, but often underappreciated, role in mediating reproductive development in the neonatal boar (Figure 8). Androgens made by perinatal Leydig cells appear to be important for the modulation of sexual behaviors postnatally. For example, after cessation of the perinatal surge in testosterone production, the incidence of pre-pubertal mounting behaviors increased in boars, but not in gilts or males castrated at birth (Berry and Signoret 1984). Moreover, neonatally castrated boars treated with estrogen after puberty displayed female sexual behaviors (Ford 1982, 1983b), whereas the lordosis response was sparsely stimulated by 17β-estradiol treatment in males castrated later (6–8 mo) in life (Ford 1982; Ford and Christenson 1987). Female sexual behaviors were also infrequent in neonatally castrated boars supplemented with testosterone during development (Ford and Christenson 1987). These data demonstrate a clear role for perinatal testosterone production in the regulation of male sexual behaviors.

Growing evidence also suggests that perinatal sex steroids regulate Sertoli cell proliferation. For example, reducing endogenous estrogen production via administration of a CYP19 inhibitor (letrozole) in the neonatal period promoted Sertoli cell proliferation; accordingly, the testes of letrozole-treated boars were 25% larger and sperm production was enhanced (At-Taras et al. 2006; Berger et al. 2008). These effects were not mediated by alterations in LH secretion, suggesting direct effects of reduced 17β-estradiol synthesis (At-Taras et al. 2006). Similar results were observed in a subsequent study that used the estrogen receptor antagonist, ICI 182,780 (Berger et al. 2013). A previous report also suggests that androgens may directly modulate Sertoli cell proliferation; neonatal use of flutamide also increased Sertoli cell numbers (Legacki et al. 2015). Thus, elevated neonatal testosterone levels appear to be important regulators of Sertoli cell proliferation in the porcine testis.

Interestingly, neonatal androgen production by perinatal Leydig cells may also be important for the development and function of adult Leydig cells. For example, neonatal boars treated with flutamide from PND 2–10 had fewer, hypertrophic Leydig cells in adulthood. In addition, histological analyses revealed that flutamide-treated boars had clusters of Leydig cells with abnormal morphology (Kotula-Balak et al. 2012), which may be groups of peritubular Leydig cells first described by Van Straaten and Wensing (Van Straaten and Wensing 1978). In addition, treated boars also produced less serum testosterone, but 17β-estradiol levels were greater due to increased production of CYP19 (Kotula-Balak et al. 2012). However, gonadotropin secretion was unaffected, suggesting a testis-specific effect (Kotula-Balak et al. 2012).

### Androgens in the adult boar

#### Spermatogenesis

Testosterone has many important roles in reproductive function of the adult boar (Figure 8). One of the most well-recognized is the vital role of testosterone as a mediator of spermatogenesis. After production by Leydig cells, the majority of testosterone diffuses into the seminiferous tubules to support spermatogenesis (Walker and Cheng 2005); 20% of testosterone is released into the blood in the boar (Setchell et al. 1983). In fact, testicular testosterone levels are 50- to 100-fold higher than in peripheral blood (Walker and Cheng 2005). The mechanism that concentrates testosterone within the tubular compartment is not well understood in the pig. Androgen binding protein (ABP), which concentrates androgens within the seminiferous tubules of other species (Ritzen et al. 1975), has not been detected in swine (Cook et al. 1977; Jegou and Le Gac-Jegou 1978).

Androgens are thought to affect somatic cells of the porcine testis, including peritubular myoid, Leydig, and Sertoli cells (Zhou et al. 2002). Within the seminiferous tubules, androgens are thought to act primarily on Sertoli cells, which express the androgen receptor (AR) in a stage-dependent manner (Zhou et al. 2002). Endocrine and genetic ablation experiments suggest that androgens are most important in the stages of spermatogenesis when AR is highly expressed (stages VI–VII) in Sertoli cells (Zhou et al. 2002). Conflicting results exist in the literature, but, in general, the AR is thought to be absent in germ cells of most species (Sar et al. 1990; Bremner et al. 1994; Zhou et al. 2002). In one study utilizing pigs, the AR immunolocalized to only somatic cells of the testis (e.g., Leydig, Sertoli, and peritubular myoid cells). In other studies, however, AR was detected in porcine germ cells (Ramesh et al. 2007) and on mature, ejaculated spermatozoa (Rago et al. 2007). These discrepancies remain unresolved; therefore, androgens may also directly affect germ cell development and/or function in the pig.

Androgens appear to control the onset of spermatogenesis since high doses of testosterone induced spermatogenesis in pre-pubertal primates (Marshall et al. 1984); likewise, sperm is detectable in the seminiferous tubules closest to androgen-producing Leydig cell tumors in boys (Weinbauer and Nieschlag 1996). Conversely, androgen deficiency or loss of AR function in post-pubertal animals results in the arrest of spermatogenesis and infertility (Cattanach et al. 1977; Sharp 1994; Quigley et al. 1995; Chang et al. 2004). While little information is available in the pig, Sertoli cell-specific knockout of AR in mice demonstrated that androgens are required for three different phases of spermatogenesis: 1) the progression of germ cells through meiosis; 2) the transition from round to elongated spermatid; and 3) spermiogenesis (Chang et al. 2004; De Gendt et al. 2004; Holdcraft and Braun 2004). Indeed, germ cell degeneration begins during stage VII of spermatogenesis in the absence of testosterone (Sharp 1994). Moreover, postmeiotic spermatogenesis was ablated in mice lacking the AR DNA binding domain within Sertoli cells (Lim et al. 2009).

The biological mechanisms by which androgens regulate spermatogenesis are not well defined, especially in the pig. In the mouse, treatment with testosterone upregulated 234 testicular genes and downregulated about twice as many within 24 h. However, few genes known to be associated with spermatogenesis were identified (Sadate-Ngatchou et al. 2004). In another study, androgens were implicated as important mediators of tight junction assembly between adjacent Sertoli cells (Meng et al. 2005). Indeed, AR expression in Sertoli cells is greatest when premeiotic germ cells migrate from the basal to adluminal compartment of the seminiferous epithelium, which occurs through the remodeling of tight junctions (Russell 1977; Bremner et al. 1994; Zhou et al. 2002). Androgens enhance expression of the tight junction protein, Claudin 3, which is essential to maintain the blood–testis barrier (Meng et al. 2005). Conversely, loss of AR functionality in Sertoli cells enhances the permeability of the blood testis barrier, leading to azoospermia in mice (Meng et al. 2005). In the boar, reduced intratesticular testosterone levels disrupted the function of the blood–testis barrier due to aberrant expression of tight and gap junction proteins, which ultimately impaired spermatogenesis (Kopera et al. 2011; Hejmej et al. 2012). Thus, androgens mediate spermatogenesis, at least in part, via maintenance of the blood–testis barrier.

While testosterone is essential for spermatogenesis, only a small percentage of basal levels are required. In the rat, only 4–20% of normal testosterone levels were required to maintain spermatogenesis (Rommerts 1988; Sharpe et al. 1988). Likewise, LH receptor knockout mice that maintain reduced levels of testosterone production continued to achieve post-meiotic germ cell differentiation (Zhang et al. 2003). Despite an approximately 98% reduction in intratesticular testosterone, 30–40% of men treated with testosterone (with or without gonadotropin suppression) retained fertility, demonstrating that effective male contraception requires combined suppression of both gonadotropins and androgen action (Huhtaniemi et al. 1987; McLachlan et al. 2002). Therefore, high contraceptive efficiency is only achieved with both anti-gonadotropin and anti-androgen treatments in humans (Anderson and Baird 2002). These data suggest that low levels of testosterone are required to maintain spermatogenesis in mammals. Once that threshold is met, however, further increases in testosterone concentrations do not appear to yield greater sperm production. For example, boars selected for 10 generations based on testosterone production did not have enhanced sperm output (Walker et al. 2004).

#### Sperm maturation within the epididymis

While the testis is the site of sex steroid and sperm production, the epididymis is the critical organ for sperm maturation (Brooks 1983). As sperm leaves the testis, it enters the epididymis, which has three regions through which sperm transit (caput, corpus, and cauda, respectively). During this transit, immature sperm interact with localized epididymal secretions (e.g., proteins) to gain motility and fertilization capacity (Brooks 1983; Dacheux et al. 2005; Pearl et al. 2007). Importantly, steroid hormones produced in the testis (and transported primarily through luminal fluid) are critical regulators of these epididymal secretions (Pearl et al. 2007). Indeed, at least half of the epididymal proteins secreted in boars are under androgen control (Dacheux et al. 2005) as evidenced by a large reduction in epididymal protein synthesis and secretion following castration (Syntin et al. 1999; Ezer and Robaire 2003). This is supported by the expression of AR in both principal and basal cell nuclei in all three epididymal regions (Pearl et al. 2007). Interestingly, ERβ, but not ERα, was also expressed in both cell types throughout the epididymis (Pearl et al. 2007), suggesting a synergistic regulatory role for both androgens and estrogens in epididymal secretions. In fact, when castrated boars were supplemented with testosterone alone, pre-castration protein secretory levels in the epididymis were not achieved (Syntin et al. 1999).

New evidence suggests that androgens also regulate the production of extracellular vesicles within the epididymis, termed epididymosomes (Suryawanshi et al. 2012; Rodgers et al. 2013; Zhang et al. 2021). Extracellular vesicles are natural nanoparticles within body fluids that transmit bioactive cargo (e.g., non-coding RNAs) from donor cells to recipient cells (O’Brien et al. 2020). During epididymal transit, immature sperm receive bioactive cargo from epididymosomes, which directly promotes sperm maturation (Sharma et al. 2018). In addition, non-coding RNAs delivered to sperm by epididymosomes have been implicated in mediating epigenetic inheritance and offspring health. Although data in swine remain limited, boar epididymosomes represent an emerging area of interest (Rodriguez-Martinez et al. 2022).

#### Accessory sex gland function

It has been known for over a century that the accessory sex glands of the boar require factors produced by the testis to develop (Griffiths 1897). Early studies demonstrated that the accessory sex organs of a barrow (prostate, seminal vesicles, bulbourethral gland) are small, fibrous, and poorly developed compared with specimens from the boar. Logically, the secretory capacity of the accessory sex glands diminishes post-castration. In one report, both ejaculate volume and gel production quickly (within 1 wk) decreased following castration (Joshi and Raeside 1973). In addition, citric acid and fructose levels in seminal plasma, which are indicators of androgenic activity (Banks et al. 1956), also declined after castration in pigs (Joshi and Raeside 1973). Supplementary treatment with testosterone and estrogens were required to fully rescue the secretory activity of accessory sex glands (Joshi and Raeside 1973). In a different study, treatment of barrows with androgens increased the weight of the prostate, seminal vesicles, bulbourethral glands and submaxillary glands (Booth 1980). Histological analysis revealed that the increased weight was due to the development of secretory epithelium and the accumulation of fluid (Booth 1980). Treatment with exogenous steroids also restored fructose and zinc levels, both indicators of androgen stimulation, within the seminal vesicles of castrates (Booth 1980). In other species, androgens have also been implicated in regulating the production and cargo of extracellular vesicles from accessory sex glands (Sullivan and Saez 2013), but data in swine remain limited.

#### Sexual behavior

Testis-derived steroids are the primary mediators of sexual behavior in the pig (Hemsworth and Tilbrook 2007). For example, castration reduced copulatory behavior within 30–60 d in sexually experienced boars (Joshi and Raeside 1973; Levis and Ford 1989). Likewise, muscles of the penis and the urethra were underdeveloped in the barrow (Griffiths 1897). Testosterone as well as its metabolites, 17β-estradiol and DHT, appear to synergistically mediate sexual behavior in the boar. Treatment with DHT alone, which cannot be aromatized to estrogen, failed to fully rescue sexual behaviors in barrows (Parrott and Booth 1984; Levis and Ford 1989). Likewise, treatment of castrates with 17β-estradiol initiated, but did not sustain, normal sexual behaviors (Parrott and Booth 1984). In another study, treatment with estrone only partially (55%) restored latency to first mount in barrows (Joshi and Raeside 1973). However, co-treatment of 17β-estradiol and DHT fully restored sexual behaviors (Levis and Ford 1989). Likewise, treatment with testosterone, which can be aromatized to 17β-estradiol or reduced to DHT, fully restored the sexual behavior of barrows (Joshi and Raeside 1973; Levis and Ford 1989) or boars immunized against GnRH-I (Esbenshade and Johnson 1987). So, both androgens and estrogens are required for sexual behavior of the boar (Levis and Ford 1989).

While androgens and estrogens are required for sexual behavior of the male pig, evidence suggests that only threshold concentrations are necessary for maximal display of copulatory behaviors; hormone levels above this threshold do not enhance the display of reproductive behaviors (Hemsworth and Tilbrook 2007). Conversely, suppression of testosterone secretion does not fully ablate sexual behavior in boars (Estienne et al. 2004). Thus, enhanced libido is not due to elevated testosterone levels (Hemsworth and Tilbrook 2007). Therefore, it has been proposed that the variation in libido between animals with sufficient testosterone levels is due to differential responsiveness to testosterone in tissues that mediate reproductive behaviors (Grunt and Young 1952). Reduced sexual interest, however, may be due to decreased production of testicular steroids if the threshold is not met (Hemsworth and Tilbrook 2007). Therefore, a dramatic reduction in steroid production, like castration, is often necessary to impair male sexual activity (Hemsworth and Tilbrook 2007). However, the effectiveness of castration on decreasing reproductive behaviors is dependent on age at castration and sexual experience of the animal, suggesting that sexual behaviors can be learned (Hemsworth and Tilbrook 2007).

### Estrogens in the adult boar

The pig testis produces very high levels of estrogen compared to other species (Velle 1958), reaching almost 280 pg/mL in the blood (Claus and Hoffmann 1980). Serum estrogen concentrations are even greater in boars than in females during estrus (Claus et al. 1985). Boars also have elevated levels of 17β-estradiol in the seminiferous tubules and in semen (Claus et al. 1985). Approximately 85% of the estrogen in porcine seminal plasma originates from Leydig cells, whereas 15% is produced from the accessory sex glands (Claus et al. 1985). Interestingly, spermatozoa are carriers of estrogen in boar semen and may help accumulate steroids within the seminiferous tubules (Claus et al. 1985) since ABP is not produced in the boar (Cook et al. 1977; Jegou and Le Gac-Jegou 1978). As previously discussed, estrogens are thought to work synergistically with androgens to aid in accessory sex gland function (Joshi and Raeside 1973). Further, research suggests that estrogens also have a distinct role in boar fertility.

In the mature boar, CYP19 activity occurs exclusively in the Leydig cells (Corbin et al. 1999; Ziegler et al. 1999; Kotula-Balak et al. 2012), which supports previous data demonstrating that steroids are produced solely outside of the seminiferous tubules in the boar testis (Setchell et al. 1983). Indeed, porcine Leydig cells, but not Sertoli cells, secrete estrogens in culture (Raeside and Renaud 1983). The major estrogen produced by the boar testis is estrone (Velle 1958). Estrogen receptors (ER) are nuclear receptors found in many cell types in the boar reproductive tract, including Leydig cells, Sertoli cells, spermatogonia, spermatocytes, and epididymal cells (Ziegler et al. 1999; Wagner et al. 2006). In fact, ERs were detected in all three regions of the epididymis (caput, corpus, and cauda), suggesting a potential biological significance (Pearl et al. 2007). Moreover, ERα and ERβ have been identified in mature, ejaculated porcine spermatozoa (Rago et al. 2007); interestingly, porcine sperm also expresses CYP19 (Rago et al. 2007), suggesting that testosterone can be converted to 17β-estradiol in seminal plasma. Ultimately, these data suggest that estrogens play a role in spermatogenesis, sperm maturation, and sperm function in the pig.

Several studies confirmed that estrogen is needed for sperm development in the boar. For example, boars immunized against GnRH-I had 60% fewer spermatogenic cells (except A-spermatogonia) and a 50% reduction in mitosis of spermatogonia (Wagner et al. 2006). After infusion with 17β-estradiol, however, spermatogenic cell numbers and mitosis partly recovered (Wagner et al. 2006). In addition to spermatogenesis, estrogen appears to play a role in sperm maturation and capacitation. For example, researchers examined the effects of treating boars with the CYP19 inhibitor, letrozole, on sperm maturation. Compared to controls, letrozole-treated boars had altered caput and corpus epididymal sperm morphology, as well as a reduction in the number of sperm overall and the number of sperm capable of fertilization within the cauda epididymis (Pearl et al. 2007). Exogenous estrogens also appear to be important for sperm capacitation in the pig. For instance, treatment with estrogens enhanced sperm capacitation (Ded et al. 2010). In another study, xenoestrogenic compounds also promoted sperm capacitation and the acrosome reaction in porcine spermatozoa (Mohamed et al. 2011).

In addition to aiding in the development of mature sperm, estrogens also appear to assist in fertilization in the pig. Interestingly, the estrogens present in seminal plasma play a role in fertilization by aiding in sperm transport and ovulation in the sow (Claus et al. 1989; Claus 1990; Claus and Schams 1990). For example, estrogen infusion (near physiological levels) into the cervix increased the frequency of myometrial contractions 2.5-fold through stimulating the release of prostaglandin F2α. Consequently, the number of spermatozoa bound to the zona pellucida was increased in females that received intracervical 17β-estradiol prior to artificial insemination (Weitze et al. 1990). Together, these data demonstrate that estrogen production in boars is an important component to porcine fertility.

## Conclusions

In the boar, Leydig cells are uniquely specialized and drive a broad spectrum of reproductive processes spanning fetal sex differentiation, neonatal programming, spermatogenesis, sperm maturation, accessory sex gland function, and sexual behavior. Unlike most species, boars exhibit three distinct phases of Leydig cell development and function across the lifespan. Across these developmental stages, fetal, perinatal, and adult Leydig cell populations serve distinct roles, supporting fetal sexual differentiation, neonatal endocrine programming, and adult spermatogenesis and fertility, respectively. The interstitial compartment of the boar testis is among the largest of all livestock species and is densely packed with Leydig cells, supporting the robust production of many different sex steroids. Indeed, the boar testis has been described as “the most versatile steroid-producing organ known” (Raeside et al. 2006). Compared with many other mammals, porcine Leydig cell steroidogenesis exhibits several distinctive features, including prominent estrogenic capacity driven by CYP19 expression, unique steroidogenic enzyme localization and isoform expression, and retention of a functional GnRHR-II. Leydig cell development is highly plastic during the fetal and neonatal periods, and Leydig cell function is differentially regulated across the lifespan through both LH-dependent and LH-independent mechanisms, rendering this cell population potentially vulnerable to pathological disruption. Given the central role of the boar in modern swine production, elucidating the mechanisms governing porcine Leydig cell development and function across the lifespan is imperative for maximizing boar fertility and promoting reproductive efficiency in the swine industry.

## Abbreviations

17OH-P5: 17α-hydroxypregnenolone
17β-HSD: 17β-hydroxysteroid dehydrogenase
3β-HSD: 3β-hydroxysteroid dehydrogenase
ABP: androgen-binding protein
AI: artificial insemination
AR: androgen receptor
ATP: adenosine triphosphate
cAMP: cyclic adenosine monophosphate
CREB: cAMP response element-binding protein
CYP11A1: cytochrome P450 family 11 subfamily A member 1 (cholesterol side-chain cleavage enzyme; P450scc)
CYP17A1: cytochrome P450 family 17 subfamily A member 1
CYP19: cytochrome P450 family 19 (aromatase)
DHEA: dehydroepiandrosterone
DHT: dihydrotestosterone
DPC: days post-coitus
ER: estrogen receptor
ERα: estrogen receptor alpha
ERβ: estrogen receptor beta
FSH: follicle-stimulating hormone
GnRH-I: gonadotropin-releasing hormone I
GnRH-II: gonadotropin-releasing hormone II
GnRHR-I: gonadotropin-releasing hormone receptor I
GnRHR-II: gonadotropin-releasing hormone receptor II
Gs: stimulatory G protein
hCG: human chorionic gonadotropin
HDL: high-density lipoprotein
HPG: hypothalamic–pituitary–gonadal (axis)
ICI 182,780: estrogen receptor antagonist
INSL3: insulin-like 3
kDa: kilodalton
LDL: low-density lipoprotein
LH: luteinizing hormone
mRNA: messenger RNA
P450scc: cholesterol side-chain cleavage enzyme
P5: pregnenolone
PKA: protein kinase A
PKC: protein kinase C
PND: postnatal day
rER: rough endoplasmic reticulum
sER: smooth endoplasmic reticulum
StAR: steroid acute regulatory protein
Δ4: delta-4 steroidogenic pathway
Δ5: delta-5 steroidogenic pathway
